# Cardiovascular Risks Associated with Gender and Aging

**DOI:** 10.3390/jcdd6020019

**Published:** 2019-04-27

**Authors:** Jennifer L. Rodgers, Jarrod Jones, Samuel I. Bolleddu, Sahit Vanthenapalli, Lydia E. Rodgers, Kinjal Shah, Krishna Karia, Siva K. Panguluri

**Affiliations:** Department of Pharmaceutical Sciences, College of Pharmacy, University of South Florida, Tampa, FL 33612, USA; jlrodgers2@health.usf.edu (J.L.R.); jcjones@mail.usf.edu (J.J.); samuelignati@mail.usf.edu (S.I.B.); sahit@mail.usf.edu (S.V.); lerodgers47@knights.ucf.edu (L.E.R.); kinjal1@health.usf.edu (K.S.); krishnakaria@health.usf.edu (K.K.)

**Keywords:** aging, gender, cardiovascular disease, estrogen, testosterone

## Abstract

The aging and elderly population are particularly susceptible to cardiovascular disease. Age is an independent risk factor for cardiovascular disease (CVD) in adults, but these risks are compounded by additional factors, including frailty, obesity, and diabetes. These factors are known to complicate and enhance cardiac risk factors that are associated with the onset of advanced age. Sex is another potential risk factor in aging adults, given that older females are reported to be at a greater risk for CVD than age-matched men. However, in both men and women, the risks associated with CVD increase with age, and these correspond to an overall decline in sex hormones, primarily of estrogen and testosterone. Despite this, hormone replacement therapies are largely shown to not improve outcomes in older patients and may also increase the risks of cardiac events in older adults. This review discusses current findings regarding the impacts of age and gender on heart disease.

## 1. Introduction

Age plays a vital role in the deterioration of cardiovascular functionality, resulting in an increased risk of cardiovascular disease (CVD) in older adults [1,2]. The prevalence of CVD has also been shown to increase with age, in both men and women, including the prevalence of atherosclerosis, stroke and, myocardial infarction [3]. The American Heart Association (AHA) reports that the incidence of CVD in US men and women is ~40% from 40–59 years, ~75% from 60–79 years, and ~86% in those above the age of 80 [3]. Thus, older adults present a major burden for current US healthcare infrastructure, due to the high prevalence of CVD. The burden of CVD is directly related to increased mortality, morbidity, and frailty in affected individuals, which also translates to significant overall healthcare costs [3]. Given that the aged US population is expected to increase by 2050, by as much as two- and three-fold, the need for a better understanding of the etiologies associated with CVD in older adults is critically needed [3]. 

Many risk factors have been linked to the development of CVD, such as hypertension, diabetes, and obesity [4,5]. However, sex differences are also frequently observed in aging adults, with regards to both onset and prevalence of CVD [4]. In the AHA 2019 Heart Disease and Stroke Statistical Update, the incidence of CVD was reported to be 77.2% in males and 78.2% in females, from ages 60–79 years [6]. Furthermore, the incidence of CVD was reported to be 89.3% in males, and 91.8% in females, in adults above 80 years of age [6]. With respect to coronary artery disease (CAD), the strongest risk factors are male gender and age [7]. Overall, sex differences that lead to discrepancies in CVD risk factors and outcomes, between men and women, are largely attributed to sex hormones and their associated receptors [4]. Given the wide gap in cardiac risk factors between premenopausal and postmenopausal women, estrogen (E2) has been studied extensively for its potential cardioprotective activity [4]. However, hormone replacement therapies (HRT) which utilize estrogen treatment are largely controversial, due to the potential for severe side effects, with inconsistent benefits [8]. In this review, we discuss the current understanding of the impact of age in the development and incidence of CVD, in particular, a focus on gender discrepancies in CVD in aged adults, in order to provide a better of understanding of considerations needed in the development of future treatments within the aging population. 

## 2. Pathophysiology of CVD in Aged Adults

**Figure 1:**
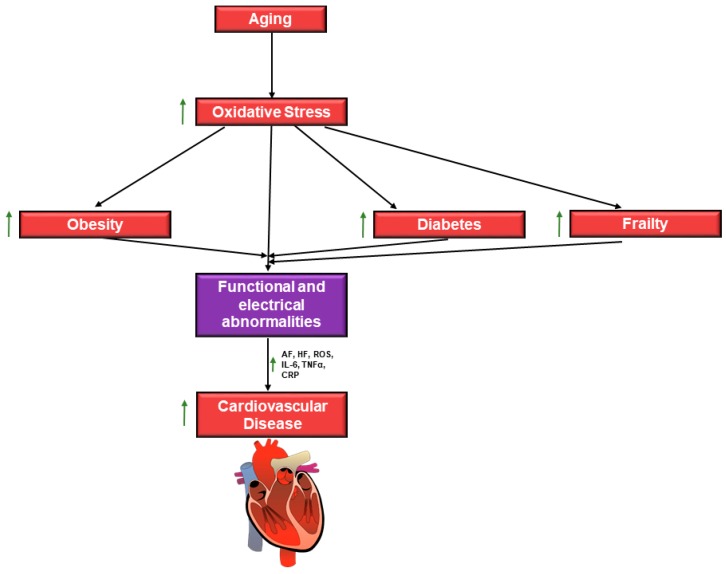
Schematic diagram of age-related risk factors for cardiovascular disease (CVD). Age is associated with increased oxidative stress, which leads to an increased susceptibility for functional and electrical abnormalities which lead to CVD. These abnormalities include atrial fibrillation (AF) and heart failure (HF), which are a result of increased reactive oxygen species (ROS) due to oxidative stress and increase production of inflammatory signal molecules. Age is also associated with an increased risk for frailty, obesity, and diabetes. These conditions are also independent risk factors for CVD. Multiple risk factors result in a high incidence of CVD in aging adults. In this figure 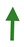 indicates upregulation. Tumor necrosis factor-α (TNFα), C-reactive protein (CRP), and interleukin-6 (IL-6).

Functional changes in aging adults hearts have been characterized, which include reports of diastolic and systolic dysfunction, and also electrical dysfunction, including the development of arrhythmias [9]. Collectively, both functional and electrical defects result in a high prevalence of heart failure, atrial fibrillation, and other CVDs, in aging patients [9]. The high prevalence of CVD in this population (Figure 1) has been linked to a number of factors, including increased oxidative stress, inflammation, apoptosis and overall myocardial deterioration, and degeneration [1]. An increase in the production of reactive oxygen species (ROS) is known to occur with the onset of advanced age [1,2], and is linked to persistent inflammation and progression to chronic disease status, as in CVD [1]. Increased production of proinflammatory markers is a hallmark of aged hearts, including high levels of interleukin-6 (IL-6), tumor necrosis factor-α (TNFα), and CRP (C-reactive protein) [1]. Production of inflammatory factors and other mediators contribute to cardiac remodeling, including significant extracellular matrix (ECM) remodeling, which is caused by impaired ECM turnover [1,10]. Dysregulation in matrix metalloproteinase (MMP) and tissue inhibitor of metalloproteinase (TIMP) expression levels are frequently linked to increased collagen deposition and the development of cardiac hypertrophy and fibrosis in aged hearts [10]. Fibrosis and hypertrophy are both significant structural changes that lead to eventual cardiac dysfunction in aging patients [11]. Fibrosis, due to impaired ECM turnover, has been shown to develop in the atria of aging patients, which also results in atrial fibrillation in many of these patients [12].

Oxidative stress, including the production of excess ROS that occurs with cardiac aging, will also lead to mitochondrial dysfunction [9]. Cardiac aerobic metabolism is greatly dependent on mitochondrial production of ATP; thus, the loss of mitochondrial function plays a major role in the development of cardiac dysfunction in aging adults [13]. It has been reported that mitochondrial DNA is particularly susceptible to oxidative damage, since it lacks protective histones, and is in close proximity to ROS production during electron transport [14]. ROS production has also been shown to impair the efficiency of mitochondrial respiration, which also contributes to the cardiac aging process via augmented ROS production [14]. Mitochondrial oxidative stress has also been shown to result in impaired calcium signaling via dysregulation in the type 2 ryanodine receptor (RyR2) [15]. RyR2, a calcium ion channel, is primarily responsible for the release of calcium from the sarcoplasmic reticulum, allowing for muscle contraction [15]. Decreased activity of sarcoplasmic reticulum Ca^2+^ ATPase pump (SERCA) has also been observed with age [16]. Generation of biologically active lipid mediators may also result in response to age-related inflammation. Mitochondrial dysfunction due to increased ROS has been reported to result in production of lipid oxidation, which has been linked to the development of atherosclerosis [17]. Although impaired lipid metabolism via mitochondrial dysfunction is known to occur with age, however, this process is still not completely understood. One experimental study in mice reported that diets enriched with omega-6 in older aged mice leads to chronic low-grade inflammation and impaired oxidative-redox balance, resulting in electrocardiographic disturbances [18]. Collectively, age-related oxidative stress results in significant cellular and structural changes, and these eventually lead to impaired cardiac functionality and development of CVD.

## 3. Prevalence of Cardiovascular Diseases in Aging and Elderly Adults 

Age is a significant independent risk factor for CVD, since it is associated with an increased likelihood of development of any number of other additional cardiac risk factors, including obesity and diabetes [19]. The prevalence of most types of CVDs is considerably higher among older adults as compared with the general population [6]. According to the AHA, between the years 2013–2017, 77.8% of women and 70.8% of males in the range of 65–74 years were diagnosed with high blood pressure, or hypertension [6]. Rates for diagnosed hypertension increased drastically to 85.6% in women and 80.0% in men, aged above 75 years [6]. Hypertension is a major risk factor for CVD and it has been linked to several factors such as alcohol consumption, nutrition, smoking, and obesity [20]. Among older adults, hypertension is particularly associated with age, female gender, and obesity [20]. According to the AHA, coronary heart disease (CHD) is more common in older men than in older women [6]. Heart failure with preserved ejection fraction (HF*p*EF) is more common in the elderly, and is more common in older women than in older men [21]. Regarding myocardial infarction (MI) in adults aged 60–79 years, 11.5% of men had a diagnosed MI, while only 4.2% of women were diagnosed with MI [6]. Rates of diagnosed MI are also higher in men over 80 years as compared with women [6]. However, this data does not reflect the potential discrepancies related to diagnosis of acute coronary syndrome in women, including reported underdiagnoses and misdiagnosis of cardiac events in women, including in MI [22,23,24,25].

Arrhythmias are also found to increase with age and are reported to be one of the major risk factors for sudden cardiac death [26,27]. Among elderly adults, aged 66 to 93 years old, persistent atrial fibrillation (AF) was reported in approximately 10% of the outpatient population above the age of 66 years [26]. AF generated about 1.5% of strokes in adults aged 50–59 years, and up to 23.5% of strokes in older adults aged 80–89 years [28]. According to the AHA, in those hospitalized for stroke from ages 65 to 84 years, females and males had approximately equal inpatient hospital stays, but, women ≥85 years accounted for nearly 66% of all stroke patients [6]. With the number of incident strokes projected to increase twofold over the next 40 years (2010–2050), the geriatric population (≥75 years old) are likely to experience the bulk of these potentially fatal events [28]. 

## 4. The Prevalence of Aging Adults Admitted to Critical Care

Risks associated with age present an inimitable difficulty with regards to medical treatment, especially with respect to critical and intensive care treatments. Given that the prevalence of health complications increases with advanced age, it is no surprise that the average age of patients admitted to the ICU is approximately 60 years [29]. However, advanced age has been reported to be associated with increased mortality in ICU patients, even after controlling for preexisting morbidities [30]. Thus, advanced age is a risk for mortality in ICU patients [31], regardless of treatment intensity [32]. While age is an independent risk factor for mortality in ICU patients, the presence of health conditions and diseases are known to significantly augment the risk of mortality in these patients [31]. Thus, a higher prevalence of CVD in elderly ICU patients has been reported, including higher rates of heart failure, arrhythmia, and valvular heart disease [31]. High mortality rates due to CVD in critically ill patients have even resulted in the implementation of specialized health units for cardiac patients, referred to as coronary care units (CCU), or more recently, cardiovascular intensive care units (CICU) [33].

Another risk for aging adult ICU patients is the use of mechanical ventilation. The average age of ventilated patients in the ICU is ~60 years [29]. Importantly, both age and length of ventilation are associated with mortality in ICU patients [34]. Advanced age is also an important factor associated with an increased risk of failed extubation. Studies demonstrate that approximately 35% of elderly patients are reintubated within 48 to 72h after extubation [35,36,37,38]. Ventilation with high levels of supplemental oxygen presents another potential risk for elderly patients. Supplemental oxygen is frequently implemented for the treatment of hypoxia, in order to improve arterial oxygen levels in critically ill patients [39]. However, high oxygen exposure (hyperoxia) has been also been shown to induce oxidative stress due to increased production of ROS, which results in significant lung injury [40,41,42]. Additionally, hyperoxia is known to induce hemodynamic changes, including the appearance of decreased heart rate, stroke volume, and cardiac output in patients [43,44]. Of critical concern, hyperoxia in critically ill patients is also strongly associated with increased risks for poor outcome [45,46,47,48,49,50,51,52,53] and high mortality rates [51,54,55,56,57].

Reports suggest that cardiac patients may be at a greater risk for worsened outcomes with supplemental oxygen exposure. One study reported that hyperoxia in MI patients is associated with increased infarct size, recurrent MI, and development of arrhythmias [58]. The cardiac impact of hyperoxia exposure in patients has not been well defined, but there are certain experimental reports in animal models that suggest a significant cardiac risk. Experimental reports of hyperoxia exposure have demonstrated negative cardiac effects in rabbits [59], rats [60], and mice [61,62,63,64]. In mice, the effects of high levels of supplemental oxygen have shown disparate effects in females, including higher rates of mortality and more severe repolarization defects [64]. These results suggest that special therapeutic considerations are needed for ventilation in patients with age, sex and CVD, upon admission to the ICU.

## 5. Management and Treatment of CVD in Older Adults

While age is shown to be independently associated with inflammation and a risk for CVD, health behaviors may also complicate these factors. Health behaviors that are commonly linked with poor outcomes in CVD patients include inactivity, poor nutrition, and smoking [65,66]. The AHA reported that these health behaviors, in addition to poor sleep behavior, are all associated with higher risk for development of CVD [6] Self-management of these health behaviors, under the direction of medical care specialists, have shown promise in CVD patients. Thus, lifestyle modifications are a key to promoting better health in aging adults, and are a fundamental approach to reducing cardiovascular risk in adults [67]. Examples of lifestyle changes that have been directly linked to decreased risk of CVD include maintenance of a healthy weight, avoidance of tobacco products, and regular exercise [67]. Diet supplementation with inorganic nitrate has demonstrated beneficial effects on vascular function in older adults, via improvements in endothelial function [68]. Thus, inorganic nitrates may also reduce vascular stiffness, and thus the risk of atherosclerosis [68]. Enhanced endothelial function and vascular flexibility, following mineral nitrate supplementation, has been shown to lead to an overall reduction in systolic pressure, particularly in older adults with mild hypertension [68]. Endothelial function may also be improved by the addition of dietary antioxidants [69]. Vitamin C, vitamin E, polyphenols, and carotenoids have all been shown to reduce oxidative stress, and thereby may provide a protective effect against CVD [69,70]. Antioxidants are reported to work primarily by reducing the production of ROS, typically associated with advancing age, which may help to avoid initiation of the inflammatory cascade [70]. Excess ROS results in oxidation of lipoprotein (LDL), which is also shown to result in the development of CVD [69]. Reduction of ROS by antioxidants prevents the uptake of oxidized LDL into macrophages, avoiding their conversion into foam cells, and further prevents these from adhering to the endothelium, which would otherwise result in the development of atherosclerotic lesions [69]. The use of antioxidants has also been shown to prevent the release of destructive inflammatory cytokines, which play a crucial role in the activation of the inflammatory cascade [70]. 

Physical inactivity has been reported to be a major cause of chronic illnesses, such as CVD [71]. Regarding the benefit of physical activity, walking has been reported to aid older men in the management of coronary heart disease [70]. Additionally, regular walking activity may increase longevity by decreasing the risk of CVD and other age-related diseases [72]. Additionally, exercise has been shown to be particularly beneficial to aging adults, by protecting against age-related adverse systemic and cellular effects of aging, and by reducing cellular senescence [73]. Additionally, exercise is reported to improve endothelial function in older adults, but with certain differences in males and females [74]. Specifically, endurance exercises are more consistently associated with improved endothelial function in males than in postmenopausal women, due to their lack of estrogen, and subsequently increased oxidative stress [74,75]. Telemedicine and telemonitoring has also gained exposure for its potential to prevent and/or gauge risk factors associated with CVD [76]. Studies show that self-management via mobile and telehealth technologies can improve outcomes in patients with hypertension, such as the use of mobile blood pressure monitoring [77,78]. Unfortunately, elderly adults show low participation in these technologies [77,78].

In addition to lifestyle changes, statins, a class of lipid-lowering drugs, are typically implemented as a primary measure to prevent CVD [79,80,81]. Statins have been reported to reduce total cholesterol in older adults [72]. Statin use has been shown to result in a 31% decrease in low-density lipoprotein (LDL) cholesterol, and a 14% increase in high-density lipoprotein (HDL) cholesterol, in older adult patients [72]. Statins are associated with decreased all-cause mortality and cardiovascular events in older individuals without an established CVD diagnosis [72]. Overall, statins lower the risk of MI and stroke in older adults [72]. Administration of statins in older adults with diagnosed CVD is reported to result in a 14% decrease in triglyceride levels [72]. Moreover, in one study statins were found to decrease the risk of MI by 39.4% in older adults, as compared with those treated by a placebo [72]. Furthermore, statin treatment has also lead to a 23.8% decreased risk of stroke as compared with a placebo [82]. Although statins are the primary medications for atherosclerosis patients, this class of medications is responsible for muscular dysfunctions, myopathy, rhabdomyolysis, and elevated creatinine kinase levels [83]. Thus, the risks of this medication in older adults must be carefully examined when considering its overall benefit. Additionally, while statins have been reported to reduce cardiac mortality in patients after MI, one study reported lower rates of reduction for women as compared with men [84]. However, a recent study has published findings which demonstrate that women are less likely to participate in recommended statin therapy after their first MI, as compared with men, which may partially account for diminished mortality reduction for women after MI [85].

## 6. Risk Factors Associated with Cardiovascular Diseases among the Elderly

In this review, we explore age as a prominent risk factor in the development of CVD. We have discussed physiological aging of the heart as a major causative factor in the onset and manifestation of CVD in aging adults, as a result of increased oxidative stress and inflammation [1,2]. However, the risk of developing CVD is associated with many factors, and not simply a consequence of aging [86,87]. Among the factors that are associated with a higher risk for developing CVD is the higher prevalence of co-morbid risk factors in these patients, including frailty, obesity, and diabetes.

## 7. Diabetes in the Elderly Population

Diabetes is a major predisposing factor for the development of CVD in the aging population [88]. Additionally, older diabetics are reported to be at an excessively higher risk for vascular complications, due to their potentially longer disease duration [89]. Currently, diabetic cardiomyopathy (DCM) is the primary cause of mortality in diabetic patients [90]. The prevalence of diabetes is very high among adults aged 65 years and over [88]. In older patients, and in the general population, type 2 diabetes is the most prevalent [91]. Specifically, among older adults aged 65 years and above, the combined prevalence of diagnosed and undiagnosed prediabetes and diabetes is between 50–80% [88]. It has also been projected that the number of patients 65 years and over with diagnosed diabetes will increase up to 4 fold by 2050 [92].

DCM describes heart disease which develops in patients primarily due to diabetes [90]. Studies demonstrate that cardiovascular complications, like stroke, ischemia, and angina, are very common in geriatric diabetic patients [93]. While heart disease is the leading cause of mortality in diabetes, and diabetics have a higher risk for mortality due to CVD as compared with nondiabetics, it has been reported that many elderly diabetic patients may be unaware that they have heart disease [89]. Additionally, one study also reported that silent ischemic events may also be more common in elderly diabetics [89]. 

Type 2 diabetes mellitus (T2DM) is known to have gender bias [94]. While the prevalence of T2DM is higher in men, the most prominent risk factor, obesity, is more common in women [94,95]. Furthermore, cardiovascular risks associated with diabetes also appear to be higher in women [90]. Diabetic women are at a higher risk for heart failure as compared with men, including a higher risk for mortality due to coronary heart disease (CHD) [96]. Additionally, mortality due to diabetic cardiomyopathy is also reported to be higher in women than in men [90]. Diabetes also appears to attenuate the protective effect of estrogen against cardiac disease in premenopausal females [94]. The cardiac disadvantage in diabetic women has been linked to lower levels of high-density lipoprotein cholesterol (HDLC) in these patients as compared with both non-diabetic women and diabetic men [94].

The risks for development of type 2 diabetes in older adults is attributed to genetics, lifestyle and other physiological aspects of aging, such as inflammation and oxidative stress [91]. These effects, in combination, can cause hyperglycemia through impaired insulin production or insulin sensitivity. In aging diabetics, poor glucose homeostasis has been shown to be more closely associated with impaired insulin secretion by beta cells, rather than due to tissue insulin sensitivity [91]. Additionally, age-related beta cell function has also been linked with certain genetic alterations [97]. Inflammation and inflammatory cytokines also play a key role in the development of type 2 diabetes [98,99,100]. Additionally, certain inflammatory markers have been shown to predict mortality due to cardiovascular disease in diabetic patients. In one study, type 2 diabetics with the highest C-reactive protein (CRP) levels were shown to have a 76% greater risk for mortality related to cardiovascular complications [100]. Given that aging and diabetes are both risk factors for vascular inflammation, a higher risk for CVD is observed for aging diabetics [101] 

Among adults aged 65 years and above, the risk of developing CVD has also been linked to fetuin-A, a hepatic secretory protein that is synthesized in the liver and secreted into blood serum [86]. Higher concentrations of fetuin-A influence the onset of CVD through increased risk of type 2 diabetes, higher body mass index (BMI), higher waist circumference, higher LDL cholesterol, as well as elevated triglycerides, cholesterol, homeostatic model assessment-insulin resistance (HOMA-IR), and C-reactive protein (CRP) levels [102]. The relationship between fetuin-A and occurrence of CVD differs between individuals with and without type 2 diabetes [102]. Those with high fetuin-A levels and type 2 diabetes, have an 18% higher risk of developing CVD as compared with their non-diabetic counterparts [103]. Overall, there is an inverse association between fetuin-A and CVD in people without type 2 diabetes [102]. 

## 8. Obesity in the Elderly Population

Age is a major risk factor for CVD, since it represents an increased likelihood of the development of other additional risk factors, including obesity [19]. Obesity, as in diabetes, has been linked to persistent inflammation and oxidative stress [104,105,106,107]. The cardiovascular risks involved with obesity are partially mediated by concurrent presentation of high blood pressure, cholesterol, and glucose [19]. However, one study in patients with high BMI found that interventions which control hypertension, hyperglycemia, and high cholesterol may only reduce the risks for coronary heart disease by half and the risk for stroke by about three-quarters [19]. These results indicate that BMI is an independent risk factor for CVD, which has also been supported by additional studies [108,109,110]. Recently, studies have found that centralized adiposity, marked by waist circumference and/or waist-to-height ratio, is a better indicator of CVD risk factors, including risks of mortality due to CVD [111,112]. One study showed that abdominal obesity in the elderly is strongly associated with the prevalence of atherosclerotic cardiovascular disease (ASCVD) [111]. Another study indicated that high waist-to-height ratio in elderly adults is strongly associated with a high cardiovascular risk [113]. Given the prevalence of obesity in adults, as high as 37.5% in men and 39.4% in women over the age of 60 years [114], high BMI and/or central adiposity present a significant risk for CVD in the aging population; the data are also reflective of the higher prevalence of obesity in women than in men. Accumulation of visceral fat in women after menopause has been linked to hormonal changes, such as decreased estrogen, which result in an increased risk of metabolic syndrome and cardiovascular complications in obese postmenopausal women [115]. Further studies indicate that the risk of BMI-associated CVD mortality is also higher in women than in men [109]. Of critical concern, the prevalence of obesity in older adults is expected to continue to increase [116]. 

## 9. Frailty in the Elderly Population

Another important risk factor associated with the development and manifestation of CVD among the elderly, is the onset of frailty [102]. Among men and women aged 65 years and over, the presence of significant frailty has been shown to accurately predict incident CVD [11]. Frailty is known to be a direct consequence of weakened physiological reserve, which results in a heightened vulnerability towards either acute or chronic illness [26]. More than 20 frailty measures have been developed, and most of these are used to assess the key phenotypic aspects of frailty, such as decreased walking speed, exhaustion, inactivity, muscle wasting and weakness [117]. Muscle wasting is commonly associated with frailty in older adults, and includes the development of sarcopenia, or significant loss of muscle mass [117]. Studies have shown that the onset of frailty is also strongly associated with a higher incidence of disability, hospital admissions, poor outcomes, and mortality [118,119]. 

In a similar way to age-related obesity and diabetes, increased oxidative stress is a risk factor for the onset of frailty in older adults [105,106,120]. Frailty, and associated sarcopenia, is a common outcome of age-related inflammation, and is also augmented in those with metabolic syndrome, including insulin resistance [117]. The frailty phenotype increases the risk of developing CVD through its positive and significant correlation with central adiposity and inflammation, which are both observed at a high prevalence in aging adults [118]. Sex has also been shown to play a role in the development of frailty in older adults. Females are more likely than men to develop frailty [121]. This may be due, at least in part, to lower baseline muscle mass in women as compared with men [121]. Additionally, one study reported that frailty in females may also be a stronger independent predictor of mortality than in males [119]. 

## 10. Sex Differences That Arise from Hormones in Aging Adults

**Figure 2:**
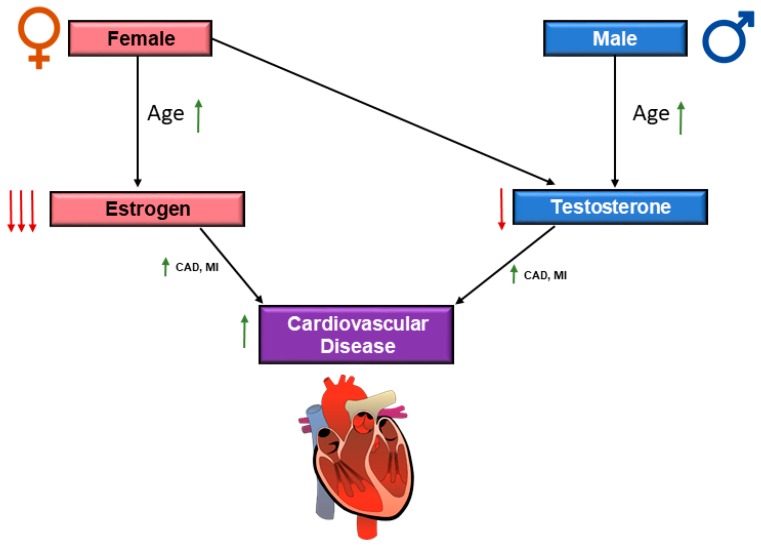
Schematic diagram of the influence of sex hormones on cardiovascular disease (CVD). Male and females both demonstrate a decline in sex hormones, while hormonal decline is more drastic in females with the onset of menopause. The decline of sex hormones is associated with increased cardiac risk in both males and females, with the decline of testosterone and estrogen, respectively. In this figure 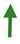 indicates upregulation and 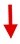 indicates downregulation. Coronary artery disease (CAD) and myocardial infarction (MI).

Within the context of this review, we have discussed several differences in CVD risk factors that are associated with sex in aging adults. While age is an independent risk factor for CVD in both men and women, it is evident that older women are more prone to certain complications which are related to heart disease [6]. Generally, before menopause, women are relatively protected from cardiovascular disease, and then, after menopause, the risk for cardiac disease greatly increases in women. The decline of sex hormones has been shown to play an important role in the development of CVD with the onset of advanced age (Figure 2), in both men and women [122]. Thus, we discuss the roles of both estrogen and testosterone in CVD in older adults. 

## 11. The Impact of Estrogen on CVD in Aging Adults 

Estrogen is often recognized for its cardioprotective role and is reported to be directly associated with the overall lower incidence of CVD in premenopausal women, as compared with age-matched men [8,122,123]. Estrogen has been shown to exert a cardioprotective effect in males as well [124,125,126]. One study reported that males are likely to develop heart disease 10–15 years earlier than women due to the gradual decline in estrogen levels after puberty [127]. Conversely, men of 70 years of age have lower overall cardiovascular risk as compared with women at age 50, the average age of menopause in women [86], which is a strong indication that estrogen decline has a greater impact on CVD risks in women than in men. It has been reported that the risk for CVD increases dramatically in women at the onset of menopause, by as much as 2–4 times [128]. In addition to increased risk for CVD, women at menopause are also at a greater risk for high LDL cholesterol levels, hypertension, diabetes, and obesity, which further elevates cardiovascular risk factors in both perimenopausal and postmenopausal women [128]. Clinical studies report a high incidence of CAD in young women who have undergone bilateral oophorectomy provide further support of the likely cardioprotective role of estrogen, since the most abundant form of estrogen, 17beta-estradiol, is secreted primarily by the ovaries [129]. Experimental studies in mice have also reported the cardioprotective effect in both males and females, via mechanisms which include those which support mitochondrial homeostasis and reduced oxidative stress [8,130,131]. Other experimental studies in mice have supported the effects of estrogen and include mechanisms which promote neo-angiogenesis and those that prevent fibrosis [8]. 

Because of the heightened cardiovascular risks in women after menopause, estrogen replacement therapy has been studied as a potential therapy for CVD. However, to date, the effects of estrogen replacement on cardiovascular health is still largely controversial [8]. The Nurse’s Health Study published epidemiology data from ~28,000 postmenopausal women, with no history of CVD, and demonstrated that estrogen therapy was associated with reductions in CVD incidence and mortality, by 40% and 50%, respectively [128]. However, a 20-year follow-up study, in this population, demonstrated that estrogen therapy was correlated with an elevated risk for stroke [132]. Further controlled trials have shown that estrogen replacement therapy is largely associated with increased cardiovascular risks in older women (>67 years) with diagnosed CVD [133]. Furthermore, the Heart and Estrogen/Progestin Replacement Study (HERS) was stopped after only four years due to an increased incidence of MI and death in women undergoing hormone replacement, which was presumed to be due to the higher risks associated with CVD in the older female population [133]. More recent research suggests that estrogen therapy in women, after the onset of menopause, may also be the cause for many of the clinical trials involving hormone replacement [8]. There have been reported cardioprotective benefits in women when estrogen replacement is introduced in early menopause, suggesting that there may be a “critical window” for estrogen replacement therapy [134,135]. More study is needed in order to determine if this critical window therapy may also provide a reduced cardiovascular risk for women at older age as well. 

## 12. The Impact of Testosterone on CVD in Aging Adults 

Testosterone, the major sex hormone for men, is also demonstrated to exert cardioprotective function [136]. In men, low testosterone levels due to hypogonadism is correlated with advanced age and other factors, such as obesity [137]. Epidemiological studies report an increased risk for cardiovascular disease in aging adult men that is associated with hypogonadism, including reduced levels of testosterone [138,139]. Low testosterone was also shown to be independently associated with a high risk for acute MI in type 2 diabetic males [140], and a high incidence of coronary artery disease (CAD) in men [7]. In older men, low testosterone levels have been linked to a higher risk for stroke [141]. An additional study reported that men, at 40 years of age, that had serum testosterone levels below the recommended threshold level, also had a higher mortality risk due to CVD [142]. Since testosterone levels show a decline in both men and women with advanced age, the impact of testosterone in postmenopausal women has also been studied. Subsequently, it was found in one study that low levels of testosterone were also associated with coronary artery disease (CAD) in postmenopausal women [143]. 

Epidemiological studies which have demonstrated a link between low testosterone levels and an increased risk for cardiovascular events, have led to studies involving testosterone replacement as a potential therapy. However, the benefits of testosterone therapy in hormone deficient men with or without pre-existing cardiovascular disease is largely controversial [137]. One clinical trial reported an increased risk for MI, stroke, and mortality with testosterone therapy [144], while another trial suggested that testosterone protected men against all of these same risks [145]. In a larger meta-analysis study that reported results from approximately 3000 older men, it was found that men treated with testosterone had a 54% increase in cardiovascular events, as compared with those treated with a placebo [146]. A larger retrospective study, with results from 56,000 individuals, it was demonstrated that testosterone therapy in men is linked to a 36% higher risk of MI within three treatments [147]. As of 2019, the US Endocrine Society (ES) reported that there is not sufficient current evidence to accurately assess the cardiovascular effect of testosterone replacement therapy [137]. Thus, the ES recommends caution when implementing testosterone therapy in older men with CVD, and further recommends against the use of testosterone replacement in men who have had a cardiovascular event within 6 months [137]. Currently, the ES primarily recommends testosterone therapy for the treatment of pathological hypogonadism in men, such as in disorders involving the hypothalamus, pituitary, and testes [137]. While a majority of men show diminished testosterone levels with advanced age, these levels are still considered within the normal physiological range, without symptoms of androgen deficiency and hypogonadism [148]. However, a small proportion of older men (1–2%) will show evidence of androgen deficiency, including testicular dysfunction, and various sexual and physical symptoms [137]. Testosterone therapy for 12 months in this population of older men has demonstrated benefits in sexual function and mood, and in certain aspects of vitality, such as increased walking distance [149,150]. Testosterone was also found to increase hemoglobin levels, as well as increase coronary non-calcified plaque volume [150]. It is important to note that these studies did not report major cardioprotective benefits in men with testosterone therapy, and similarly did not report any major adverse effects in the heart [149,150]. Despite the potential benefits of hormone replacement therapy in older men, the US Endocrine Society does not currently recommend testosterone therapy in asymptomatic older men (>65 years), particularly as an “anti-aging” treatment, due to the lack of complete evidence for the potential long term risks of hormone therapies, as in potential cardiovascular risks [137]. 

## 13. Conclusions

Cardiovascular disease (CVD) is a major health concern in the aging population. While age is an independent risk factor for CVD, other additional risk factors that are closely associated with advanced age, have been shown to compound these risks, including frailty, obesity, and diabetes. Overall, although females have a longer life expectancy than males, women make up the most significant percentage of CVD diagnoses in the elderly population, or in those greater than 80 years old [117]. These comorbid risks, in combination, are shown to compound cardiovascular risk factors in older patients, upon admission to the ICU. Gender is yet another major risk factor regarding the onset, manifestation, and management of CVD in aging adults. The decline in hormone levels may play a significant role in the development of CVD in older men and women, but hormone replacement therapies have not yet shown a significant benefit in older adults, with respect to cardiovascular health. Given that the number of older patients will continue to increase, it is critically necessary to uncover the impact of hormones on cardiovascular risk factors, in future clinical and research studies, in order to improve outcomes in the older population.
